# The Role of Traditional Leafy Vegetables on Household Food Security in Umdoni Municipality of the KwaZulu Natal Province, South Africa

**DOI:** 10.3390/foods12213918

**Published:** 2023-10-26

**Authors:** Mjabuliseni Simon Cloapas Ngidi

**Affiliations:** 1 Department of Agricultural Extension and Rural Resource Management, School of Agricultural, Earth and Environmental Sciences, College of Agriculture, Engineering and Science, University of KwaZulu-Natal, Private Bag X01, Scottsville, Pietermaritzburg 3201, South Africa; Ngidim@ukzn.ac.za; 2 Centre for Transformative Agricultural and Food Systems, School of Agricultural, Earth and Environmental Sciences, College of Agriculture, Engineering and Science, University of KwaZulu-Natal, Private Bag X01, Scottsville, Pietermaritzburg 3201, South Africa

**Keywords:** food (in)security, traditional leafy vegetables, HFIAS, ordered logit model

## Abstract

This study assessed the effect of traditional leafy vegetables (TLVs) on the level of food security in the rural area of Dlangezwa, in the KwaZulu-Natal province of South Africa. A total of 100 respondents were selected using a simple random sampling method. The Household Food Insecurity Access Scale (HFIAS) was used to measure the food security status. The influence of TLVs on household food security was examined using an ordered logit model. Pumpkin leaves and blackjack were the most consumed traditional leafy vegetables in the study area, at 97% and 81%, respectively. The results from HFIAS showed that 17% of the households were food secure, 44% were mild food insecure, 26% were moderately food insecure and 13% were severely food insecure. The results from the chi^2^ test showed that there was a significant correlation between the intake of cowpeas (*p* = 0.02), blackjack (*p* = 0.01), and moringa (*p* = 0.02) with the degree of household food security in the surveyed houses. The ordered logit model’s findings indicated that consumption of leafy vegetables, educational attainment, employment status, and marital status all had a substantial impact on the food insecurity of the households. The marital status of a household showed a positive and significant impact on the food insecurity situation, while educational level, employment status, and consumption of leafy vegetables showed a negative and significant impact. For enhanced household food security, there is a need for initiatives that encourage the use of a wide range of traditional leafy vegetables such as Moringa oleifera, Amaranthus, and cowpeas. More awareness should be made regarding the nutritional benefits that TLVs have.

## 1. Introduction

South Africa is listed among the countries with the greatest levels of absolute poverty and economic inequality in the world [1]. In South Africa, the Gini coefficient is thought to be 0.68 [2,3]. Nearly 28 percent of South Africa’s population lives in extreme poverty, below the food poverty level [4,5,6]. South Africa may be food secure at a national level, but a sizable portion of households and individuals are food insecure [4,7,8]. In the KwaZulu-Natal province and South Africa in general, household socioeconomic position, as shown by income, job status, and food expenditure, is highly correlated with household food insecurity [9]. Similarly, the province of KwaZulu-Natal and its Umdoni Municipality are faced with similar challenges. In order to achieve food security, total household income is crucial [10,11]. However, due to the high rate of poverty in the province and municipality, it is challenging for most households to buy enough food to feed their entire household throughout the entire year. In order to face the challenges related to poverty and food insecurity, traditional leafy vegetables (TLVs) can play an important role in ensuring household access to nutritious food in the province, country and Africa in general [12].

Traditional leafy vegetables are defined as any plant species that are either native to a given location or that have been present there for such a long time that they have undergone natural or farmer-selected evolution [12]. Traditional leafy vegetables are crucial for rural households’ ability to maintain food and nutrition security. TLVs give household members a supply of nutritious food and improve their resilience to food and nutrition insecurity. However, these traditional leafy vegetables are frequently ignored, and households substitute nontraditional vegetables like kale and rapeseed oil for the traditional ones. Traditional leafy vegetables are easy to grow and do not cost much to produce. Additionally, TLVs require less agricultural input than exotic vegetables because they are adapted to the local climate [13]. TLVs come in a variety of varieties, including Amaranth (*Amaranthus* spp.), cowpeas (*Vigna unguiculata*)*,* jute mallow (*Corchorus olitorius*), spider flower, pumpkin leaves, and cowpea seeds [14]. Many regionally used crops have been supplanted as the mainstream meals have grown in popularity, yet are too costly for households to produce.

Goals two and three of the Sustainable Development Goals (SDGs), which are part of the UN’s sustainable development agenda, are to end world hunger and guarantee overall health and well-being, respectively. However, one of the biggest difficulties of the twenty-first century is proving to be how to provide the world’s population with a diet that is healthful, nutritionally adequate, economical, and environmentally sustainable. In order to provide the population with healthy diets, the importance of traditional leafy vegetables cannot be ignored. Sidelining traditional leafy vegetables reduces the food options for households. Traditional leafy vegetables are high in nutrients, so growing and eating them could improve this generation’s dietary problems [15].

TLVs have long been a staple source of food for rural households. In KwaZulu-Natal, which includes Umdoni Municipality, TLVs are included in households’ diets, especially in the rural regions, but they are not heavily marketed, and there is less empirical support for their role in ensuring food and nutrition security. Several studies have been conducted on the consumption of traditional leafy vegetables [16,17,18,19]; other studies [12,14] considered the importance of TLVs as a source of food for rural households. Generally, studies [8,10,11] have not investigated the role of TLVs on household food security. There is, therefore, a need for quantitative research linking TVLs to food security indicators to offer empirical-based evidence of the role the TVLs play in reducing food insecurity and hunger-related incidences. It is against this backdrop that the study is set to determine the contribution of TVLs to households’ food security in the Umdoni Municipality of the KwaZulu-Natal province in South Africa.

## 2. Materials and Methods

### 2.1. Description of Study Area

The study was conducted in Dlangezwa, Umdoni Municipality in KwaZulu-Natal (KZN), which is located in the northeast of the province [20]. Umdoni Municipality consists of 19 wards with a geographical area of 994 square kilometers [20]. The Umdoni Municipality has three major uses of land, which includes commercial agriculture, traditional authority, and coastal urban nodes.

The Dlangezwa area normally receives about 906 mm of rain per year, with most rainfall occurring during mid-summer. It receives the highest rainfall (128 mm) in January and the lowest rainfall (18 mm) in July. The monthly distribution of average daily maximum temperatures shows that the average mid-day temperatures for the Dlangezwa area range from 22.4 °C in July to 27.8 °C in February. The area is the coldest during July, when the temperature drops to 9.7 °C on average during the night [20]. The main agricultural activities that can be found in the area include vegetable, crop, and some fruit farming, and in some cases agricultural activities include sugarcane production [20].

### 2.2. Data Collection Method

The study used a quantitative approach to collect data from farming households in 2019. Households were asked to provide demographic information and food security-related information, including the types of food crops available, grown, consumed, and sold. From the list of food crops provided by households, five traditional leafy vegetables were observed, isolated, and considered for the study. These traditional leafy vegetables included pumpkin leaves, blackjack, cowpeas, moringa, and Amaranthus. Primary data were collected through the use of questionnaires. The sample size determination was calculated using the 95% confidence interval and 5% margin of error based on the sampling frame of 257 traditional leafy vegetables farming households, with each household having an equal chance of being selected. A total of 100 respondents were selected using a simple random sampling method. A household head or acting household head responded on behalf of the entire household. Prior to being asked to complete the questionnaire, the respondents were made aware that participation was completely optional and that they could leave the study at any time. The information that respondents submitted would be kept private and used only for educational purposes. Ethical clearance (approval number: HSS0287/018) was sought and received from the Human Social Sciences Research Ethics Committee through a broader Sustainable and Healthy Food System (SHEFS) research group application. Additionally, participants were also given an informed consent form to sign before interviews to ascertain their agreement to participate in the study.

### 2.3. Data Analysis

The quantitative data were analysed using STATA statistical software (version 13) and Statistical Software for Social Sciences (SPSS) version 24. The descriptive statistics were obtained to provide the key socioeconomic characteristics of the sampled farming households. A chi^2^ (χ2) test was used to test for significant differences between the observed distribution of the data among the categories and the expected distribution at the 5% level of significant association [21].The food security status of the surveyed farming households was determined using food security categories as per the Household Food Insecurity Access Scale (HFIAS) tool. The HFIAS score measured the degree of food access challenges at the household level. It was calculated by adding the households’ responses to nine questions asking about the frequency of certain behaviors that indicate increasing challenges in accessing food in a particular household [22]. The higher scores indicate more food access challenges, while low scores indicate fewer food access challenges. The lower bound of the score is 0, while the upper bound is 27.

The validation of the Household Food Insecurity Scale involved various steps. Questions were reviewed with a group of key informants and then refined with a small group of respondents before the pretest. The pretest was conducted to ensure that the questions were clear and understandable. It was also performed to obtain community approval. While the questions in the generic questionnaire are worded to be as universally relevant as possible, certain questions contain phrases that were adapted to the local context to ensure that respondents knew their meaning.

The ordered logit regression model was used to analyze the impact of the consumption of traditional leafy vegetables on the food security of the surveyed farming households. The ordered logit model (OLM) was originated by Clogg and Shihadeh [23]. This model allows the slope coefficients to vary for each binary regression. It is used when the dependent variable (HFIAS) has two or more categories, and the values of each category have a sequential pattern [24]. The HFIAS in this study has four categories (food secure, mildly food insecure, moderately food insecure, and severe food insecure), as stated in Coates et al. [25]. Empirically, OLM is commonly employed in determining ordinal survey data [24].

The dependent variables in this case (HFIAS categories) were merged into a binary variable which took the value of 1 if the household was food secure and 0 otherwise. The three food insecurity categories were all captured as food insecure. The general logistic model may be written as follows:

(1) $$Q_{i}=f\left(y_{i}\right)=\frac{1}{1+e^{-\left(\varphi +\sum \Psi_{i}\;X_{i}\right)}}$$

where $Q_{i}$ is the probability that an individual is food insecure given $x_{i}$, $x_{i}$ represents the *i*th explanatory variables, and φ and Ψ are regression parameters to be estimated. *e* is the base of the natural logarithm. Table 1 shows the different types of independent variables.

## 3. Results and Discussion

### 3.1. Socioeconomic Characteristics of Household

According to the study’s findings, 59% of the 100 household heads examined were headed by women and 41 percent by men (Table 2). According to this study, 67% of the household heads polled were single, while 26 percent were married, and only 7% had other sorts of relationships. Only one respondent was colored, and 96% of the household heads examined were made up of members of the African ethnic group. Only 9% of the household heads in the poll reported having no formal education, while 54% of the household heads reported having a secondary education. A total of 34% of the household heads reported having only a primary education. Only 3% of the population had completed tertiary education.

The participants’ ages varied from 18 to a maximum of 83 years. The average age was 39 years. The survey population’s households ranged in size from one to eighteen. The average number of people in a household was seven. As seen in Table 2, roughly 30% of the households that were interviewed had jobs, whereas 70% of the members were unemployed. From the 30% of people who claimed being employed, it was revealed that 18% of homes belonged to farm laborers, while 12% had other jobs. The community’s primary source of income comes from the agricultural industry.

Social grants, which were received by 68% of the sample’s households, were one of the primary sources of income. One-fourth of the homes questioned (24%) had a full-time worker. In total, 6% of the households in the poll had farming as a source of income, and 6% had part-time jobs as their only source of income. Only a small percentage (2%) of the households in the study received money from nonagricultural sources, and none of the homes received income via remittances.

The monthly food budget for the household ranged from less than or equal to ZAR 1000 to more than ZAR 1000. About 80% of the households polled said they spent less than or equal to ZAR 1000 on food each month, while 20% said they spent more than ZAR 1000. The results showed that 91% of the households questioned made monthly purchases less than or equal to ZAR 1000, while 9% of respondents said they spent more than ZAR 1000 a month on other needs.

The households polled spent a bigger percentage of their monthly income on food purchases. This shows that buying food is their top priority and that they spend the majority of their monthly income on it. In general, a black family of four in South Africa pays approximately ZAR 3500 for an average food basket per month [26]. Since most households in the research region spent ZAR 1000 a month on food, they do not represent the usual South African household pattern in that they spend less than the required amount of ZAR 3500, and their household sizes varied from 1 to 18, as shown in Table 2. The possibility exists that the households’ food supply comes from community and household gardens as well as from the wild.

**Figure 1:**
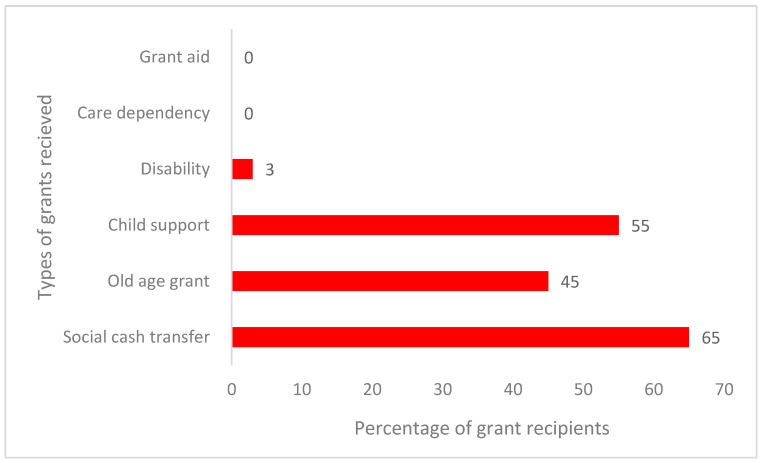
Grants received by household heads in Dlangezwa, 2019. Note: one household may be a recipient of more than one type of grant.

Figure 1 shows that grants for old age were given to 45% of respondents and social cash transfers to 65% of the household heads questioned. A total of 3% of respondents received a disability grant, while none of the respondents received care dependency or grant in aid. Child support was paid to 55% of the studied households. Most household heads received social cash transfers, grants for the elderly, and grants for child support. The respondent’s monthly income was significantly influenced by these grants.

### 3.2. Types of Traditional Leafy Vegetables Consumed

**Figure 2:**
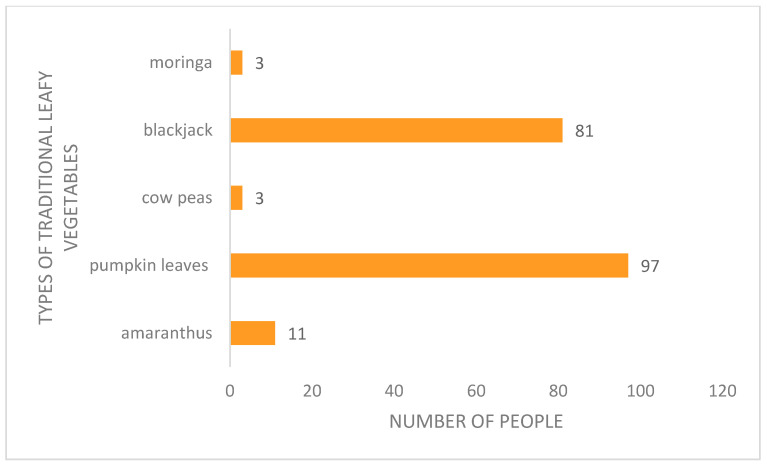
Types of traditional leafy vegetables consumed in Dlangezwa, 2019.

According to Figure 2, almost 97% of the population who were questioned consumed pumpkin leaves, and 81% consumed blackjack. Due to their year-round availability, pumpkin leaves and blackjack were the traditional leafy vegetables that the people ate the most. Since they could be found in the wild as well, no labor was needed. Due to taste preferences and them not being available year-round, just 3% of the studied households ate cowpeas and moringa. Due to the cultural knowledge that had been instilled in the respondents, they did not consume all varieties of traditional leafy vegetables. Traditional leafy vegetables are not consumed frequently because they have a taste that is frequently slightly harsh and because they are not visually appealing to consumers [15]. Amaranthus was only consumed by 11% of the studied homes because it was thought to be a Nigerian dish by the locals.

### 3.3. Household Food Security Status of Surveyed Households

The majority of polled households (75%) expressed concern that their homes did not have enough food to eat in the four weeks before the survey was conducted, according to the HFIAS survey. This showed that the majority of the households in the study experienced food insecurity. A total of 35% of respondents were concerned that their family members had gone to bed hungry in the four weeks before the survey’s administration. A third (33%) of the households were concerned that their members could spend a day and a night without eating. This suggests that a small percentage of the households surveyed experienced moderate to severe food insecurity.

The households’ issues with food access are identified by the HFIAS classification. Table 3 shows that 17% of the households questioned were food secure, meaning they had access to enough food with sufficient nutritious content to eat every day. Many of the households in the survey (or about 44%) experienced mild food insecurity. Additionally, it was discovered that 26% of the households in the study had moderate food insecurity, which implies that the quantity or quality of the food consumed was degraded due to a lack of funds to buy food or old age. Approximately 13% of the households in the survey were classified as extremely food insecure, which means that they skipped meals, went without food for days, or cut back on their food intake.

### 3.4. The Contribution of Traditional Leafy Vegetables to Household Food Security

Traditional leafy vegetables, which are consumed more rarely than other staple foods, came in 20th place in the National Food Consumption Survey (NFCS) on food consumption patterns among children aged one to nine in KwaZulu-Natal [18]. Even so, the results of the chi^2^ test indicated that there was no discernible difference in terms of food security between Amaranthus consumers and non-consumers. This was demonstrated by the study’s findings, which are shown in Table 4, which indicated that between 82 and 18% of respondents—those who did not eat Amaranthus—were determined to have adequate access to food. In contrast, the findings revealed that 9% of Amaranthus consumers and 9% of non-consumers experienced food insecurity. The most likely reason could be related to the potential for some South African provinces to outlaw consuming Amaranthus due to income from its commercialization and production [19]. In general, the households’ degree of food security was unaffected by eating traditional leafy vegetables. However, there was a significant correlation between the intake of cowpeas (*p* = 0.02), blackjack (*p* = 0.01), and moringa with the degree of household food security in the surveyed houses.

Studies on cowpeas as traditional leafy vegetables have shown a considerable contribution to dietary needs and food security [14]. However, the study’s findings were different from those of the earlier research. This revealed a substantial difference between those who ate cowpeas, a typical leafy vegetable, and those who did not in terms of their level of food security. Table 4’s findings revealed that 12 percent of respondents who ate cowpeas and 88 percent of those who did not were in a food-secure situation. However, the findings revealed that just 1% of cowpea consumers and 99% of non-consumers were food insecure, respectively. The most likely explanation is that people are aware of the therapeutic and nutritional benefits of these classic leafy vegetables.

### 3.5. Empirical Estimates of TLVs’ Contribution to Household Food Security Status

Table 5 lists the parameter estimates for the ordered logit model. The computed coefficients are not marginal effects since the ordered logit model is not linear. Marginal impacts were therefore quantified and discussed. The outcome of the VIF test for multicollinearity demonstrates that multicollinearity was not a problem, and the heteroskedasticity issue is eliminated by the insignificant *p*-value of the Breusch–Pagan/Cook–Weisberg test. The estimated model’s pseudo R-squared is 0.55. In the ordered logit model, four of the nine predicted coefficients are significant.

The sampled households’ level of food insecurity is favorably influenced by the marital status of those households, and this influence is statistically significant. The conclusion implies that there is a 2.6 unit rise in family food insecurity for every unit increase in marital status (moving from the single to married category). This might be explained by the fact that married households need to provide food for extra members. Therefore, based on these results, married households are more likely to experience food insecurity in the research area. This finding is consistent with that of Zulu et al. [27], who found that marital status had a negative influence on the acceptance of leafy vegetables, leading to less consumption of leafy vegetables, which also resulted in food insecurity. On the contrary, Zondi et al. [28] found a positive relationship between marital status and food security status of indigenous crop farmers. The authors explained that married households make informed joint decisions about the production of indigenous crops, thus resulting in improved food security status.

The study’s findings indicate a negative relationship between household food insecurity and education level. The findings show that eating traditional leafy vegetables reduces family food insecurity by 4.4 units for every unit increase in household-head education. These results imply that the consumption of native fruits increases when household-head education rises. These results lend credence to the idea that higher education might encourage the consumption of native foods since more health-conscious consumers would do so. This, however, defies the widely held belief that higher levels of education place people in higher socioeconomic strata who are more frequently connected with Western lifestyles and eating preferences at the expense of native foods [16]. Ngidi et al. [29] found that education level had a negative influence on the consumption of indigenous leafy vegetables; however, it was positive and statistically significant on the food security variable. The authors explained that as the household member becomes more educated, they tend to consume more nutritious food from various food groups, such as vegetables, fruits, grains, and protein foods.

The level of food insecurity in the home was negatively and statistically significantly correlated with employment status. This suggests that when all the other factors in the model are maintained constant, the family food insecurity lowers by 1.1, as the work status of the household head increases. These results indicate that an increase in the number of households who are working can improve the food security status of the household, with the employment status of the household appearing to reduce the food insecurity status. This is because when households receive income from their employment, they are able to purchase other leafy vegetables or other food group that they cannot produce. This supports the findings of Arene and Anyaeji’s study [30], which showed a beneficial relationship between the household head’s work status and the likelihood that the HFIAS will decline, hence improving the status of the household’s food security. Ngidi et al. [29] found that receiving a salary from an employer had a negative impact on the consumption of indigenous crops; however, it had a positive impact on food security. The authors explained that the more the salary increases from being employed, the more the household buys foods from various food groups in the market, such as vegetables and fruits and proteins, thus eradicating malnutrition and achieving food security within the members of the household.

The study’s findings indicate a negative and statistically significant link between consumption of leafy vegetables and food insecurity. This means that as the households consume more leafy vegetables, food insecurity decreases, which leads to improved food security. This is consistent with studies by Legwaila et al. [31] and Mavengahama et al. [32], which found that consuming TLVs can decrease food insecurity in low-income households by promoting dietary diversity and providing essential nutrients. The findings are also in line with those of Hunter et al. [33] and Kepe [34], who claimed that TLVs can increase household food access by allowing one to supplement diets because they can be used in place of goods that must be purchased, particularly for low-income households that find it difficult to buy food. However, the results of this study have demonstrated that wild foods are consumed by households with difficulties accessing food and with low dietary diversity as a coping strategy, echoing the findings of Mojeremane and Tshwenyane [35], Jman Redzic and Nutrition [36], Quaye [37], and Fentahun and Hager [38], and possibly serving as a “safety net” in these households [39,40].

## 4. Conclusions and Policy Recommendations

Traditional leafy vegetables are crucial for rural households’ ability to maintain food security. This study’s goal was to assess how traditional leafy green vegetables affected the food security situation of the sampled farming households in the Umdoni Municipality. The majority of the households were not only jobless and heavily relied on social assistance but were also food insecure. The most common sort of job in the area was farm work. The consumption of leafy vegetables, educational attainment, employment status, and marital status all had a substantial impact on the farming households’ food insecurity. There are some leafy vegetables that households do not like to consume or do not consider edible. While marital status did not reduce the food insecurity levels of the sampled farming households, education level, employment status, and consumption of leafy vegetables improved the food security situation of these households. Reintroducing traditional leafy vegetables to the South African diet may be the answer to most of the nation’s food and nutrition security problems. Traditional leafy vegetables should be given serious consideration as one of the pathways to reduce food insecurity in South Africa. The study recommends that households need to be encouraged to include more leafy vegetables in their diet to reduce food insecurity. There is a strong need to provide nutrition education, through specific leafy vegetable nutrition programs, on the nutritional benefits that can be obtained from different types of leafy vegetables such as Moringa oleifera, Amaranthus, and cowpeas. To solve the issue of high unemployment and heavy dependency on social assistance, nutrition programs and workshops can also be used to demonstrate different ways in which households, particularly in rural settings, can benefit financially from the sales of leafy vegetables.

## Figures and Tables

**Table 1:** Definitions and summary statistics of independent variables in Dlangezwa, 2019.

Variable Name	Variable Definition
Age	Age of the household member (in years)
Gender	Gender of the household head (1 = male, 0 = female)
Marriage	Marital status of the household head (1 = married, 0 = otherwise)
Household size	Number of family members (continuous)
Level of education	Level of education of the head in years (continuous)
Employment status	If the household is working (1 = yes, 0 = no)
Source of income	Income received by households
Occupation type	Type of employment a household is involved in

**Table 2:** Showing demographics (gender, marital status, ethnic group, and highest educational level) of the households in Dlangezwa, 2019.

Participants	Percentage (%)
N = 100	
Gender (%)	
Male	41
Female	59
Marital status (%)	
Single	67
Married	26
Other	7
Ethnic group (%)	
African	96
Other	4
Highest education level (%)	
None	9
Primary	34
Secondary	54
Tertiary	3
Employment status (%)	
Employed	30
Unemployed	70
Occupation type (%)	
Farm worker	18
Other	12
Sources of income (%)	
Full-time employment	24
Part-time employment	6
Social grants	68
Farming	6
Remittance	0
Nonagricultural enterprise	2
Nothing	11
Monthly expenditure on food: ZAR	
≤1000	80
>1000	20
Monthly expenditure on other necessities: ZAR	
≤1000	91
>1000	9

**Table 3:** Food (in)security status of the selected households in Dlangezwa, 2019.

Food Security Status of Respondents (%)	
Food secure	17
Mildly food insecure	44
Moderately food insecure	26
Severely food insecure	13

**Table 4:** Relationship between consumption of different types of traditional leafy vegetables and household food security status in Dlangezwa, 2019.

	Food Secure	Food Insecure	*p*-Value
Amarathus			
Nonconsumer	82.35	90.36	0.336
Consumer	17.65	9.6	
Pumpkin			
Nonconsumer	0	3.61	0.426
Consumer	100	96.39	
Cowpeas			
Nonconsumer	88.24	98.8	0.02 **
Consumer	11.76	1.2	
Blackjack			
Nonconsumer	47.06	13.25	0.001 ***
Consumer	52.94	86.75	
Moringa			
Nonconsumer	88.24	98.8	0.02 **
Consumer	11.76	1.2	

Notes: *** and ** indicate significance at 1% and 5%, respectively.

**Table 5:** Ordered logistic regression of contribution to household food security status in Dlangezwa, 2019.

HFIAS_Category	Coef.	St. Err.	*p*-Value	dydx
Gender	1.695	1.561	0.277	0.245
Age	−0.044	0.094	0.641	0.456
HH_Head	−2.115	1.891	0.263	0.342
Marital status	2.551	1.104	0.021 **	0.016 **
Educational level	−4.413	1.787	0.014 **	0.034 **
Race	−22.342	2058.767	0.991	0.641
Employment status	−1.139	0.474	0.016 **	0.025 **
Main source of income	−0.121	1.820	0.947	0.8454
Consumption of leafy vegetables	−8.781	5.063	0.083 *	0.063 *
cut1	−59.037	4117.551		
cut2	−48.093	4117.538		
cut3	−45.364	4117.537		
Mean dependent var				
Pseudo R-squared	0.553			
Chi^2^	44.621			
Prob > Chi^2^	0.000			
Akaike information criterion (AIC)	64.117			
Bayesian information criterion (BIC)	83.734			
VIF	4.23			
Breusch–Pagan/Cook–Weisberg test for heteroskedasticity	0.876			
Chi^2^(1)	1.44			
Prob > Chi^2^	0.230			

Notes: ** and * indicate significance at 5% and 10% level, respectively.

## Data Availability

The data presented in this study are available on request from the corresponding author.
